# Intravenous Immune Globulin Uses in the Fetus and Neonate: A Review

**DOI:** 10.3390/antib9040060

**Published:** 2020-11-04

**Authors:** Mahdi Alsaleem

**Affiliations:** 1 Pediatrics Department, Neonatology, Children’s Mercy Hospital, Kansas City, MO 64108, USA; malsaleem@cmh.edu; 2 Pediatrics Department, University of Kansas, Wichita, KS 67208, USA

**Keywords:** immunoglobulins, fetus, neonates, sepsis, hemolysis, hyperbilirubinemia, necrotizing enterocolitis, coronavirus, coronavirus disease 19 (COVID-19)

## Abstract

Intravenous immune globulin (IVIG) is made after processing plasma from healthy donors. It is composed mainly of pooled immunoglobulin and has clinical evidence-based applications in adult and pediatric populations. Recently, several clinical applications have been proposed for managing conditions in the neonatal population, such as hemolytic disease of the newborn, treatment, and prophylaxis for sepsis in high-risk neonates, enterovirus parvovirus and COVID-19 related neonatal infections, fetal and neonatal immune-induced thrombocytopenia, neonatal hemochromatosis, neonatal Kawasaki disease, and some types of immunodeficiency. The dosing, mechanism of action, effectiveness, side effects, and adverse reactions of IVIG have been relatively well studied in adults but are not well described in the neonatal population. This review aims to provide the most recent evidence and consensus guidelines about the use of IVIG in the fetus and neonate.

## 1. Introduction

**Figure 1:**
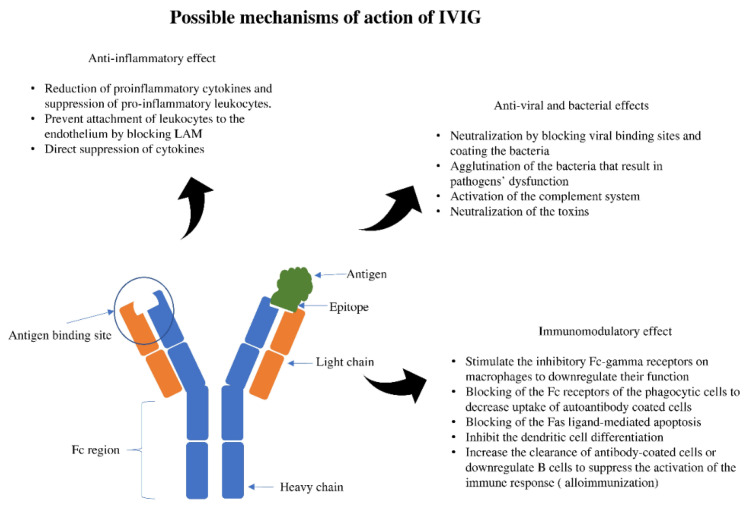
Antigen-antibody binding and specific effects. LAM; leukocyte adhesion molecule.

Immunoglobulin therapy is defined as the use of a combination of antibodies obtained from healthy human donors to treat different conditions [1,2]. The principal components of intravenous immunoglobulin (IVIG) are IgG antibodies, which compromise about 90% of the IVIG. Antibodies are glycoproteins synthesized and secreted by plasma cells (activated B cells) to respond to antigenic stimulation with the primary purpose of a specific immune response to result in different physiological and/or pathological processes [2,3]. The basic structural unit is primarily formed by two heavy and two light chains [4,5]. The difference between the heavy chains results in different kinds of antibodies: IgG, IgA, IgM, IgE, and IgD. After synthesis, formed antibodies functions by binding with a specific antigen epitope. This binding subsequently results in specific actions that ultimately help neutralize and inactivate the pathogenic organisms or trigger a specific immune response (Figure 1).

IVIG clinical applications and indications have expanded rapidly in recent years [6,7]. Several of these clinical applications have extended to include children, neonates, and fetuses [8,9]. Although the Food and Drug Administration (FDA) has not yet approved IVIG therapy for use in the neonate, it has been used off-label in the management of challenging and progressing conditions in many fetuses and neonates [9]. The roles that IVIG may play in immunomodulation (inhibition or activation of the immune response, modulation of FcgR expression on B cells, inducing phagocytosis or direct cytotoxicity, regulating apoptosis, modulation of antigen-presenting cells) were the driving factors to study the use of IVIG in this population subset [10,11].

The use of human serum in the scientific field has been reported as early as the 19th century [12]. Before the mid-20th century, most IVIG uses revolved around the management of infectious diseases [13,14,15]. The use of immunoglobulin isolated from the human serum in non-infectious conditions was first reported in 1952 [16]. International collaborations were organized to investigate the use of immunoglobulins further. These collaborations’ main goals were to standardize the treatment dose, efficacy, indications, and route of administration [17].

**Figure 2:**
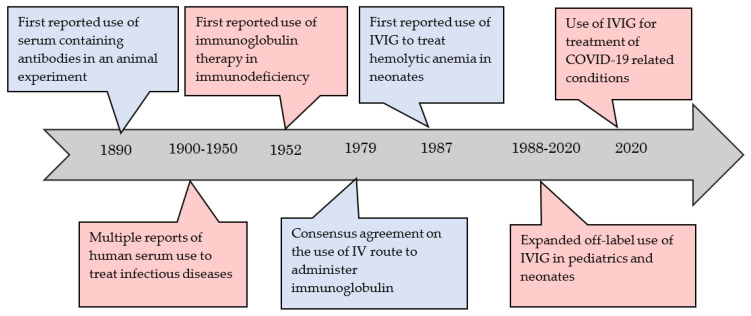
Timeline showing the important historical events in the process of immunoglobulin discovery, synthesis, and clinical applications. IVIG: Intravenous immunoglobulin.

Expanded efforts suggested using the intravenous formulation in the management of specific conditions in the non-adult population [18,19]. The first use of IVIG in neonates was reported in 1987 by Hara et al., who used IVIG to treat an infant with hemolytic anemia due to Rh incompatibility [20]. Since that time, clinical use and application in neonates and fetuses have increased significantly, and investigators have attempted to search for the best evidence for use, safety, and adverse effects. Recently, tremendous effort has been placed on the role of IVIG therapy to treat complications related to Coronavirus-19 viral infection in adults as well as the pediatric and neonatal population. A snapshot of important events in immunoglobulin therapy history is shown in Figure 2 [21].

Despite the strong evidence and the clear indications for using IVIG in adults and its clinical applications in the pediatric population, the evidence is less clear regarding neonates [22,23,24,25,26]. A summarized list of suggested clinical indications for IVIG use in the neonatal population is shown in Table 1. As this research area has been active for the past 40 years, this review highlights the practical aspects and the most recent evidence about IVIG use in the fetal and neonatal population.

## 2. Clinical Use in Fetuses and Neonates

### 2.1. Alloimmune Hemolytic Disease in Neonates

Alloimmune hemolytic disease (AIHD) of the newborn, otherwise known as the newborn’s hemolytic disease, is considered the most common cause of hemolytic disease in the neonatal period [27,28,29,30]. AIHD is regarded as the first and the most common indication for IVIG to prevent severe hyperbilirubinemia that may require an exchange transfusion [31,32]. This condition’s primary pathophysiology is due to the hemolysis of neonatal red blood cells by *maternally-derived* IgG antibodies [33]. These antibodies are derived in the maternal blood during pregnancy or shortly after when the incompatible fetal antigen enters the maternal circulation. Once these antibodies are produced, there is a potential for transplacental transfer from the maternal circulation to the fetal blood; this transfer leads to the possibility of antigen-antibody interaction that ultimately may result in hemolysis [34]. The primary two types of AIHD are ABO (The major human blood group system) incompatibility and RhD (Rhesus factor D) hemolytic disease. In the ABO form, hemolysis occurs due to existing antibodies. For example, mothers with A and B blood groups only produce IgM, which does not cross the placenta, and isoimmunization does not occur. In type O mothers, the antibodies are predominantly IgG, which crosses the placenta and can cause hemolysis in the fetus. Unlike Rh, ABO disease can occur in first pregnancies because anti-A and anti-B antibodies are found early in life from exposure to A- or B-like antigens present in many food items and other natural substances. Further, hemolysis is less severe in ABO because A and B antigen is expressed by other cell types such as endothelial cells, thus diluting the effect of circulating antibodies. On the other hand, RhD hemolytic disease usually happens during birth or pregnancy. When the mother is exposed to the paternally derived RhD antigen for the first time, IgM antibodies are formed. However, due to their relatively large size, IgM antibodies do not cross the placental barrier. Therefore first pregnancies are usually not affected. During subsequent pregnancies and repeated exposure, IgG antibodies are formed, and they can potentially transfer via the placenta and attack fetal red blood cells and ultimately result in their breakdown [35]. RhD hemolytic disease used to be the most common cause of severe alloimmune hemolysis in neonates. However, the emergence of antenatal Rh (D) prophylaxis and the intrauterine intervention offered to the affected fetuses resulted in a significant decrease in the occurrence and the severity of hemolysis associated with Rh (D) hemolytic disease [36,37]. Due to this decrease in incidence, hemolysis due to ABO incompatibility became more common; however, only about 15% of the affected pregnancies with ABO incompatibility will develop hemolysis. Only a smaller percentage will develop severe hyperbilirubinemia [38,39]. In the presence of hemolysis unexplained by either RhD or ABO incompatibility, investigation for other minor blood groups (Duffy, Kell, P, and others) or different Rh antigens (E, C, and c) incompatibility is recommended.

The clinical presentation of AIHD can affect the fetus and/or the newborn at various severities based on the hemolysis degree. Severe hemolysis during pregnancy can result in hydrops fetalis (severe anemia, resulting in heart failure and fluid accumulation in different bodily cavities (pleural effusion, skin edema, pericardial effusion, or ascites)) [40,41]. Rates of morbidities and mortality in fetal hydrops are high and may warrant intrauterine intervention to perform a fetal blood transfusion [42]. In other cases of mild or moderate hemolysis, anemia and associated hyperbilirubinemia in the neonate are the most common clinical manifestations.

Significant efforts have been made to understand and to manage the hyperbilirubinemia associated with AIHD in recent years [43,44]. The main aim of most of the studies and clinical trials has been to provide efficient, timely, and aggressive interventions to prevent the devastating complications of hyperbilirubinemia (acutely known as acute bilirubin-induced encephalopathy and long-term disabilities, and permanent neurodevelopmental deficits also known as kernicterus) [30,43,44,45]. The primary etiology of the brain damage in these two conditions is the penetration of bilirubin through the blood-brain barrier and eventually deposition in the central nervous system [46].

IVIG has been proposed as a potential intervention that can decrease hemolysis severity and, therefore, the associated hyperbilirubinemia [47,48]. The exact mechanism of the action of IVIG to reduce hemolysis is unclear. Scientists suggest IVIG works most likely by blocking the antibodies’ receptors located on the red blood cells’ surface. Blocking these receptors will prevent the antigen/antibody interactions between the antigens found on the red blood cells and the maternal antibodies, decreasing recognition of the targeted red blood cells by the circulating macrophage and subsequently decreasing the degree of hemolysis [32,49]. The first reported use of IVIG in AIHD of the newborn was published in 1987 [20]. This report was followed by other case reports and case series that suggested using IVIG as a useful intervention to halt severe hyperbilirubinemia [50,51,52]. This intervention’s primary beneficial effect is to decrease the need for exchange transfusion (a high-risk procedure performed in advanced intensive care units to prevent the risk of bilirubin-induced brain damage).

The American Academy of Pediatrics (AAP) [53], in their report in 2004, recommended the use of IVIG for alloimmune hemolytic disease of the newborn if the serum bilirubin level continues to rise despite intensive phototherapy or approaches the levels for which exchange transfusion is required [54]. The dose suggested is 500 mg^−1^ g for each kg of body weight given via the intravenous route to be infused over two hours. The AAP used the evidence obtained from the systemic review performed by Gottstein et al., and other previous observations that showed the beneficial effects and the favorable outcomes after using IVIG to manage severe hyperbilirubinemia [20,32]. The AAP also advised using IVIG in the rare types of Rh disease (Anti-C and Anti-E) but acknowledged the limited evidence behind this recommendation [54].

Supported by these recommendations by the AAP, there was a significant increase in IVIG use in severe hyperbilirubinemia due to AIHD. A recent Cochrane review by Zwiers et al. was done in 2018, to further evaluate this practice’s evidence-based aspects [55]. In their meta-analysis, 27 full-text articles were screened for eligibility. Nine studies were eligible, and a total of 658 participants were included for the final analysis. The results did not support the AAP’s recommendations. They concluded that there was not enough evidence that IVIG use in AIHD prevents exchange transfusion. In their conclusion, the authors recommended using IVIG if performing exchanging transfusion is not possible at the admitting facility until a transport arrangement can be made to a higher-level center.

More recently, two studies were performed to evaluate the efficacy of IVIG. El Fekey et al. found in their randomized controlled trial that the use of IVIG in addition to phototherapy resulted in a decrease in bilirubin levels and the number of exchange transfusions performed [56]. In contrast to these findings, Al-lawama et al. found in their retrospective observation that infants who received IVIG in addition to phototherapy were noted to be at higher risk for rebound hyperbilirubinemia and the need for exchange transfusion [57]. However, both of these studies were limited by the small sample size and confounding variables’ effects.

Louis et al. did a systematic review and meta-analysis that included 12 studies about the safety and efficacy of IVIG in neonates with RhD hemolytic disease. They found after analyzing the data based on high vs. low risk of bias that IVIG is beneficial in RhD hemolytic disease of the newborn in studies with a high risk of bias; however, this benefit was not clear in the studies that carried a low risk of bias (evaluated by risk assessment including reviewing; appropriate randomization, allocation, completing the outcome data, selective reporting, and others) [58].

Such conflicting outcomes could be explained by the different response to IVIG therapy based on the primary etiology. De Haas et al. and Armstrong et al. suggested in their report that IVIG may be more effective if the hemolysis is due to ABO groups incompatibility vs. RhD incompatibility [59,60]. Another possible explanation may be related to the origin and the characteristics of the specific IVIG formulations used in the different studies.

One randomized double-blinded placebo-controlled trial was conducted to address the use of IVIG in hemolytic disease of newborns as a prophylaxis measure rather than treatment. The subjects were infants affected by hemolysis due to Rh disease. A total of 41 infants out of the 80 included in the study received IVIG as a prophylactic measure to prevent the need for exchange transfusion. Seven infants in the intervention group required an exchanged transfusion compared to 6 from the control group. Therefore, the authors concluded the prophylactic IVIG did not significantly decrease the need for exchange transfusion in infants with alloimmunization due to Rh hemolytic disease [61].

### 2.2. Neonatal and Fetal Alloimmune Thrombocytopenia

Fetal and neonatal alloimmune thrombocytopenia (FNAIT) is thrombocytopenia caused by maternal-fetal antiplatelet antibodies, resulting in platelet destruction [62,63]. The most commonly accepted theory for pathophysiology is maternal IgG antibody formation against fetal paternally derived antigens. These IgG antibodies can pass through the placenta and subsequently form an antigen-antibody complex that ultimately results in platelet destruction.

Many platelet antigens have been identified as potential triggers for this immune process. Human platelet antigen (HPA)-1a is considered the most common trigger for maternal antibody formation and hence fetal and neonatal thrombocytopenia [64]. FNAIT usually affects the first-born child more than subsequent children, in contrast to AIHD, which affects subsequent pregnancies with a more severe degree of hemolysis [63]. Postnatal clinical manifestations vary significantly. More than half of the cases are asymptomatic and identified mainly by screening complete blood count laboratory evaluation for other reasons. Severe thrombocytopenia can lead to rapid progressive bleeding. The most feared complication is spontaneous intracranial hemorrhage (ICH) [65,66].

The main principles of treatment of FNAIT are anticipation, antenatal IVIG therapy, postnatal recognition, and timely interventions. Risk anticipation is based on the maternal history of a previous child who developed thrombocytopenia during the second or the last third of gestation or shortly after delivery due to FNAIT in previous pregnancies. The mothers identified have a risk for subsequent deliveries with potential risk for ICH,

Multiple studies have addressed the benefits of antenatal management of FNAIT. Bussel et al. found in their prospective study that IVIG administration in mothers who had a history of infants affected by FNAIT resulted in a significant increase in fetal platelet counts. None of the neonates had ICH [67]. A recent meta-analysis was done by Winkelhorst et al. to evaluate the effect of IVIG in the management of FNAIT. Four randomized trials and 22 nonrandomized observations were included in the analysis; however, pooling for statistical analysis was not feasible due to the significant heterogenicity. The study found that IVIG treatment with or without the addition of corticosteroids at different dosing regimens is a reasonable approach when considering antenatal management to prevent the risk of bleeding in the affected neonates [66]. Risk stratification and suggested dosing regimens are shown in Table 2 [66,68]. The justifications for aggressive management once affected mothers are identified are the high rate of recurrence in subsequent pregnancies and the need to prevent ICH’s devastating outcome.

To evaluate the intervention from a different perspective, Rossi et al. surveyed mothers who were treated with IVIG for FNAIT to address the significant potential interference with maternal lifestyle. The surveys were sent to 62 mothers. Of the 32 mothers who responded, 24 (75%) reported a negative influence on their lifestyle due to the treatment frequency and the side effects. The authors concluded that further research should emphasize optimizing the dose and the frequency of administration to help alleviate some of these negative significant lifestyle interferences and the physical and mental burden of IVIG treatment for FNAIT [69].

Based on the studies mentioned earlier, the use of IVIG in FNAIT is recommended [65,66,67]. However, the evidence is less clear when it comes to the postnatal management of neonatal alloimmune thrombocytopenia. Baker et al. performed a systematic review to address postnatal interventions for the Treatment of FNAIT. Fourteen articles (four prospective trials, 12 retrospective observations, and one with an unclear type of analysis) were selected for the final review. A total of 754 infants were identified. Of these infants, 147 received IVIG and other modalities of treatment (platelets transfusion and/or steroids), and 26 received only IVIG without additional interventions. IVIG administration did not show clear evidence of improving any of the outcomes evaluated, including increasing platelet counts, ICH, and mortality. However, the studies included and the pooling analysis did not adequately address the IVIG dose or IVIG’s role in particular circumstances (no response to platelet transfusion or different HPA antigens incompatibility) [70]. A more recent report by Winkelhorst et al. prospectively followed 98 live-born infants with FNAIT whose mothers did not receive antenatal prophylaxis. Eighteen infants in this cohort received IVIG. Nine of those received platelet transfusion as well. IVIG use with or without platelet transfusion was associated with a rise of the platelet count. This response was less than the other reactions seen with the other interventions trialed in the study (no treatment, only HPA-compatible platelets transfusion, random platelets transfusion, or HPA-compatible platelets given after random platelets transfusion). The interesting result was that those who were not treated with any intervention achieved higher platelet counts at five days of life than those who received IVIG, with or without platelets [71].

Another potential cause for immune-mediated thrombocytopenia is neonatal thrombocytopenia in neonates born to mothers with idiopathic thrombocytopenic purpura (ITP). Van der Lugt et al. retrospectively evaluated all the neonates born to mothers with ITP in 31 years (1980–2011) in their institution. A total of 67 infants were identified, 20 of whom (30%) had severe thrombocytopenia with a platelet count of less than 50 × 10^9^. Treatment using IVIG in those with severe thrombocytopenia after the first platelet transfusion seemed to be an effective approach to avoid multiple platelet transfusions [72].

**Table 2:** Suggested doses of IVIG for use in the fetus and neonate.

Clinical Indication	Suggested IVIG Dose	Strength of Evidence
Alloimmune hemolytic disease of the newborn	0.5–1 gm/kg per dose can be repeated if needed [53]	Recommended by AAP
Neonatal and fetal alloimmune thrombocytopenia		
Antenatal management	0.5 gm/kg/week starts at 24 weeks (standard risk) [65] 1 gm/kg/week starts at 12–16 weeks (high risk) [65]	Recommended by ACOG. Risk stratification is based on the previous history of intracranial hemorrhage in affected siblings; no siblings with ICH (standard risk), a sibling with ICH (high risk)
Postnatal management	1 gm/kg [70]	Limited evidence
Neonatal thrombocytopenia due to maternal ITP (postnatal management)	1 gm/kg/dose [72]	The best approach is to give IVIG after the first platelet transfusion if the platelet count is <50 × 10^9^/L
Neonatal thrombocytopenia due to maternal autoimmune disease	1 gm/kg/dose daily for 2 days or 0.5 gm/kg/dose daily for 4 days [73]	Limited evidence
Neonatal infections:		
Sepsis treatment	0.5 gm/kg/dose [74]	Not recommended
Sepsis prophylaxis	0.5–1gm/kg/dose [75]	Limited evidence
Enterovirus infection	750 mg/kg/dose [76]	Limited evidence
Parvovirus infection	1 gm/kg q3 weeks [77,78]	Limited evidence
Neonatal COVID-19	2 gm/kg [79]	Limited evidence
Neonatal hemochromatosis		
Antenatal management	1 gm/kg/week starts at 14–18 weeks of gestation [80]	Recommended
Postnatal management	1 gm/kg [81]	Recommended. Given immediately after double volume exchange transfusion
Primary immunodeficiency	Varies between studies to achieve Ig level of 800–1000 mg/dl [82]	Limited evidence
Neonatal Kawasaki	2 gm/kg [83]	In addition to high-dose aspirin. Evidence derived from the pediatric population

Note: The suggested dose of intravenous immunoglobulin (IVIG) for use in different neonatal clinical indications. AAP; American Academy of Pediatrics, ACOG; American College of Obstetricians and Gynecologists.

### 2.3. Neonatal Infections

#### 2.3.1. Neonatal Sepsis Treatment and Prophylaxis

The use of IVIG in adults with sepsis has been a debatable topic. Multiple systematic reviews, meta-analyses, and other trials have shown conflicting results [84,85,86,87]. The non-standardized approach, different formulations, and dosing strategies have represented significant limitations in these studies. This ambiguity about a beneficial effect in adults with sepsis applies to the neonatal population as well. Given the high incidence of sepsis in very low birth weight infants of 1.9% and the associated high mortality, a great effort has been directed toward improving the outcomes in this highly susceptible population [88,89]. One measure that has been derived from adult studies is the use of IVIG. The proposed mechanism of action of using IVIG in sepsis is based on the essential role that immunoglobulins play in opsonization and complement activation [90,91].

The International Neonatal Immunotherapy Study (INIS) collaborative group enrolled almost 3500 infants cared for by neonatologists in nine different countries in 113 hospitals. The study aimed to evaluate the use of IVIG in neonatal sepsis. The primary outcomes were death or major neurodevelopmental disabilities at two years of age. There was no difference in the mortality rate or disabilities at two years of age between the two groups. The authors concluded the study with the statement that “IVIG therapy does not affect the outcomes of suspected or proven sepsis” [92]. After the results of this well-conducted study, researchers tried to address the use of IgM-enriched IVIG to evaluate effectiveness in infants with clinical sepsis. Capasso et al. analyzed 40 infants who received IVIG enriched with IgM and a 39-infant control group who did not receive immunoglobulin therapy. The authors found that IVIG enriched with IgM resulted in a decreased short-term mortality rate compared to the control group. However, this study was limited by its retrospective nature, small sample size, and the fact that it addressed only short-term mortality [93].

Molina et al. randomized very low birth weight infants with sepsis into two groups; one received antibiotics, and the other group received IVIG at 0.5 g/kg/day for seven days in addition to antibiotics. The mortality rate was five times higher in the group that did not receive IVIG [94].

As more studies showed that IVIG might provide a beneficial response in neonates with sepsis after the INIS trial, a meta-analysis was necessary to answer this debatable question. A recent Cochrane review performed by Ohlsson et al. included nine studies with a total of 3973 subjects analyzed [74]. Multiple outcomes were evaluated in this analysis, including mortality during hospitalization, the combined outcome of death or major disability at two years of age, and length of hospital stay. After thorough analysis, the authors found that IVIG administration, including IgM enriched formulation, did not result in any beneficial effect. The authors concluded that the evidence is strong against the use of IVIG or IgM-enriched IVIG in proven or suspected neonatal sepsis and that no further research is recommended to address this topic [74].

Another meta-analysis was performed by Ohlsson and Lacy to compare the outcomes between the preterm or low birth weight infants who received IVIG prophylaxis for sepsis and those who did not receive sepsis IVIG prophylaxis. There was no difference between the two groups regarding mortality or the incidence of necrotizing enterocolitis, bronchopulmonary dysplasia, and intraventricular bleeding. However, there was a decrease in the rate of sepsis (3%) associated with those who received prophylactic IVIG (R.R.: 0.85, 95% CI (0.74–0.98) with a number need to treat of 33) [75].

Multiple explanations have been suggested for the limited effect of IVIG for prevention or treatment of neonatal sepsis, including insufficient dosing, different sepsis causative agents, the definition for sepsis, the immature immune function of the premature infants, and lower functional complement levels [95].

#### 2.3.2. Neonatal Enterovirus Infection

Enteroviruses are RNA viruses that belong to the picornaviridae family. In contrast to the mild clinical presentation in adults, neonatal infection with enteroviruses can have severe systematic involvement. 

IVIG has been suggested as a possible beneficial intervention when administered to neonates with enterovirus infection. However, the literature’s evidence is not clear about this effect and is based on only a few studies. Abzug et al. evaluated the neonatal response to IVIG in 16 neonates who developed enterovirus infection. Nine infants were randomized to receive IVIG (750 mg/kg). A mild increase in serum neutralizing titers was noted in the infants who received IVIG but did not significantly reduce the viral load in blood or urine [76]. In one case report, IVIG resulted in a good outcome in a neonate who developed disseminated infection caused by echovirus (a member of the enterovirus family) [96]. Another case series showed a promising beneficial response in two out of six infants who developed myocarditis and central nervous system (CNS) infections caused by echovirus 11 infection [97]. The most extensive study to date is the one done by Yen et al. in which data from 65 neonates with severe enteroviral infection were retrospectively analyzed. A total of 41 (63%) received IVIG, 29 of whom received it within the first 72 h of illness. The outcomes were significantly more favorable in the group of infants who received the therapy early in the disease course. However, the dose and frequency of IVIG administration were not stated in this study [98]. 

#### 2.3.3. Neonatal Parvovirus Infection

Parvovirus is a small single-stranded DNA virus. Perinatal maternal infection can result in severe consequences for the fetus, including hydrops and fetal anemia. These effects’ primary etiology are results of the viral targeting of the packed red blood cell precursors located in the bone marrow.

The evidence of using IVIG to treat neonates with parvovirus infection is minimal and only reported in the literature in two infants who were treated successfully with IVIG. One infant was treated at 15 days of life; information about dosing and frequency was not mentioned in that report [77]. The second infant was treated with IVIG 1 g/kg administered every three weeks for eight months [78].

#### 2.3.4. Neonatal Coronavirus Disease 2019 (COVID-19)

Coronavirus disease is an infectious disease caused by severe acute respiratory distress syndrome-related coronavirus 2 (SARS-CoV-2) [99,100]. At the time of this article, COVID-19 is still an ongoing pandemic that has resulted in an overwhelming number of cases worldwide. The disease mainly affects adults and tends to have severe consequences in the elderly. However, there are increasing reports of cases in the pediatric population [101,102]. COVID-19 can result in a unique vasculitis-Kawasaki-like illness in the pediatric age group. This poorly understood association usually results in multisystem inflammatory effects [103]. Neonatal cases of COVID-19 have been described recently in the literature [104]. The exact mode of transmission, the disease’s progression, and the associated morbidity and mortality remain unclear due to the low number of reported cases.

The use of IVIG has been shown by some studies to have a possible beneficial impact on adults with COVID-19 respiratory illness [105,106,107]. This effect has not been well investigated in the neonatal population [108]. Huaping et al. reported a case series of 10 neonates born to mothers with COVID-19–related pneumonia [109]. One female infant born at 34 weeks and six days of gestation developed severe symptoms most likely related to maternal COVID-19 infection. The infant’s manifestations were shortness of breath, fever, gastrointestinal bleeding, and disseminated intravascular coagulation. She responded successfully to IVIG 2 g/kg. Based on the results from this case series and another case report [79], Yuanqiang et al. suggested in their review that the use of immunoglobulins in neonates may be beneficial. However, further exploration is needed [110].

#### 2.3.5. Neonatal Congenital cytomegalovirus (CMV)

Intrauterine infection with cytomegalovirus is one of the most common infections during pregnancy. Only about 10–15% of the infected fetus with CMV will show symptoms after birth; however, there is significant morbidity and mortality seen in these symptomatic infected infants. Due to this high morbidity, scientific basic and clinical research has been directed toward evaluating and managing congenital CMV [111]. One of the possible treatment options that have been suggested is the use of IVIG. In a relatively large, randomized placebo-controlled double-blinded trial, 124 pregnant women with primary CMV infection were randomized to either received IVIG or placebo every four weeks until 36 weeks of gestation. The authors found the use of IVIG didn’t improve the newborns’ outcomes [112]. Tanimura et al. did another small clinical trial to evaluate IVIG efficacy in mothers diagnosed with primary CMV infection. IVIG with a high titer of anti-CMV antibodies failed to decrease the risk of maternal-fetal transmission [113].

### 2.4. Neonatal Hemochromatosis

Neonatal hemochromatosis (N.H.), also known as gestational alloimmune liver disease (GALD), is a rare condition that affects neonates. The pathophysiology is best explained as an immune process caused by the maternal transfer of IgG antibodies directed toward antigens located on the fetal hepatocytes [114]. The prognosis was poor for this condition, as most infants affected developed severe liver failure [115]. Traditional treatment for N.H. depended mostly on the use of chelating agents to decrease iron disposition in the hepatocytes. However, this treatment showed only mild improvement. Most of the treated infants required liver transplantation to improve survival. With a greater in-depth understanding of this condition’s pathophysiology, newer treatment modalities have focused on controlling the immune-mediated process [116].

Treatment protocols were developed to prevent the severe consequences of anticipated N.H. by intervening during pregnancy. Whittington et al. showed in a relatively large sample that IVIG administered during pregnancy to mothers who had previous infants who developed N.H. resulted in significantly better outcomes. A total of 188 pregnant women who received treatment were compared to other women with high-risk pregnancies who did not receive treatment. The final analysis showed a significant difference in outcomes, as only 30% of the untreated pregnancies resulted in healthy infants compared to 94% in the treated group [80].

Another recent report, by Okada, et al., noted a significantly favorable outcome for infants with neonatal hemochromatosis by administering IVIG to high-risk N.H. pregnancies. In their small trial, they treated a total of eight pregnancies in six women with IVIG (1 g/kg) administered at the beginning of the second trimester at weekly or biweekly dosing frequency. This regimen was continued until 18 weeks of gestation, then weekly until the time of delivery. Only three out of the eight infants born in this study developed liver dysfunction. In these three infants, the abnormalities were transient and resolved without treatment [117]. 

Another successful approach used a double exchange transfusion followed by IVIG (1g/kg) immediate administration to clear the attacking maternal antibodies. Rand et al. reported promising outcomes and significantly improved prognosis defined as survival without the need for a liver transplant. In total, 12 out of 16 (75%) infants with N.H. survived without the need for liver transplantation compared to only 23 (17%) out of 131 infants in the historical control group [81]. Further reports confirmed favorable outcomes with a similar postnatal management approach [118,119].

### 2.5. Primary Immunodeficiency

Making the diagnosis of primary immunodeficiency in neonates may be challenging in the first few months of life due to the presence of maternal antibodies in the fetal circulation [120]. However, certain conditions may manifest early in life, mainly those associated with a severe deficiency in the humoral immune response (X-linked hyper-IgM syndrome, severe combined immunodeficiency (SCID), X-linked agammaglobulinemia, and others). Treatment with IVIG in this age group remains controversial. IVIG replacement therapy is primarily indicated for those with recurrent severe or unusual infections associated with different types of immune deficiencies like hypogammaglobulinemia, common variable immune deficiency, and others.

Agammaglobulinemia, due to the complete absence of B cells, requires IVIG replacement therapy to protect against different pathogens in this critical period of life [22]. The dosing regimen and administration schedule can vary, but generally, achieving an IgG level goal of 500 mg/dl to 800 mg/dl is recommended to prevent serious complications. Usually, these levels are achieved with lifelong administration of IVIG of 400–500 mg/kg at monthly intervals [82,121].

### 2.6. Kawasaki Disease

Kawasaki disease (K.D.) is a form of vasculitis that affects the pediatric population. The pathophysiology for this condition is not clearly known. Multiple hypotheses and theories have been proposed to explain the disease process, with infectious or autoimmune triggering or both being the most widely accepted explanation [122]. K.D. disease rarely affects neonates. Only a few cases have been reported in the literature [123,124]. The low incidence in the neonatal group is possibly related to the immature immune system and the relatively high concentration of maternal antibodies in the neonatal circulation.

The treatment algorithm in neonates affected with K.D. follows the same steps involved in the pediatric population [83]. Although high-dose aspirin and IVIG (2 g/kg) usually result in complete recovery in the majority of pediatric cases, the response is less predictable in the neonatal cases [124,125].

### 2.7. Neonatal Lupus

Neonatal lupus is the description used to describe infants who are affected by maternally derived antibodies (anti-Ro/SSA and anti-La/SSB). These antibodies can be found in the maternal serum with multiple autoimmune conditions, not limited to systemic lupus erythematosus.

Congenital atrioventricular heart block (AVHB) associated with neonatal lupus carries a significant risk for morbidity and mortality. The rule of IVIG use in this condition has been studied at a different dosing regimen with and without other interventions (dexamethasone and plasmapheresis). Friedman et al. showed in their prospective open-label trial that the use of low dose IVIG at 400 mg/kg in pregnant mothers who had a previous newborn with AVHB did not prevent the recurrence in subsequent pregnancies [126]. Other small-scale trials using a combination management approach consisting of weekly plasmapheresis, IVIG every two weeks and continued soon after birth, in addition to betamethasone, have shown to be safe and effective in second-degree heart block but not in complete heart block [127]. 

In thrombocytopenia due to maternally derived antibodies seen in infants born to mothers with an autoimmune condition, IVIG use at 1 g/kg/day for two days or 0.5 g/kg/day for four days may improve affected neonates platelets counts [73].

A summary of suggested IVIG doses to use in the fetus and newborn is shown in Table 2.

## 3. Safety of IVIG Use in Neonates

As mentioned in the previous sections, although the evidence is not clear, IVIG does provide a potential treatment option for several conditions that can present in the fetal and neonatal periods. However, the use of IVIG is not-FDA approved and most often is based on off-label use after assessing the risk and benefits of IVIG administration. IVIG administration in neonates is generally safe [128]. However, rare side effects from IVIG administration have been reported, including:

### 3.1. Necrotizing Enterocolitis

(NEC) associated with IVIG has been observed more specifically in term neonates, generating a significant debate about IVIG safety in this age group [129,130,131]. Yang et al. performed a meta-analysis to evaluate this association [132]. Five original studies were included. After completing the statistical analysis, they found that infants who received IVIG for severe hyperbilirubinemia treatment were at higher risk for NEC than those who did not receive IVIG (OR: 4.54; 95% CI 2.34–8.79). Further analysis did not show a difference in the mortality rates between the case and the control groups (95% CI, 0.15–5.13). As it is well known that NEC in neonates can be multifactorial, the authors assessed the risk of bias in these studies. They found no difference in the baseline characteristics between the cases and the control groups regarding gestational age by weeks, highest total bilirubin levels, gender, small for gestational age (SGA) status, or formula feeding.

Clinicians and scientists have attempted to understand the relationship between IVIG administration and NEC. Few proposed mechanisms for this associated have been suggested; one study hypothesized that IVIG affects the intestinal blood flow; however, the authors did not show in their experiment any change in the blood flow measured immediately and at 12–18 h in the superior mesenteric or the celiac arteries after IVIG administration [133]. Another hypothesis focuses on the possible effect of IVIG on the cytokines and inflammatory response on the intestinal endothelial cells that can result in a disturbance in the immune hemostasis. This disturbance, in turn, can lead to bowel ischemia and, subsequently, bacterial transmural translocation [134]. Mesenteric vessel thrombosis and high viscosity due to IVIG administration have also been suggested as a potential risk factor that can increase the risk of NEC [135]. Another possible explanation is that the high level of bilirubin indicates a more severe hemolysis degree, which can lead to more hemodynamic disruption to the intestinal blood flow and, therefore, higher risk for NEC. However, the number and the severity of NEC episodes were not correlated with the level of bilirubin.

### 3.2. Thrombosis

Blood vessels Thrombosis after IVIG administration has been reported in the literature with an incidence of about 1–18%. Data are limited about this complication in the neonatal population. Hinson et al. reported a preterm neonate who developed inferior vena cava thrombosis after his mother received IVIG and steroids for FNAIT [136].

### 3.3. Anaphylaxis

As IVIG contains immunoglobulins pooled from thousands of individual donors, a theoretical risk exists for possible anaphylaxis, especially in newborns with IgA deficiency. However, multiple studies reported safe use of IVIG and no cases of anaphylaxis.

### 3.4. Apnea and Cardiac Arrhythmia

Cardiac and respiratory continuous monitoring during IVIG infusion in neonates is recommended to monitor vital signs and basic cardiac rhythm. Sinan, et al. reported two newborns who developed supraventricular tachycardia during IVIG transfusion. Both babies responded well to medical therapy and were converted successfully to sinus rhythm [137]. Another rare complication that can occur during IVIG is apnea [138].

## 4. Conclusions

Despite lacking FDA approval, intravenous immunoglobulin (IVIG) has been used more recently to manage different clinical conditions in fetuses and neonates. The rationale behind its use is based on the immunomodulatory, anti-inflammatory, and immune-protective effects. Continuous monitoring during and after the infusion is recommended to observe for rare adverse effects associated with IVIG use in this population subset. Different clinical practice guidelines supported the use of IVIG in neonatal autoimmune hemolytic anemia, neonatal hemochromatosis, and antenatal management of neonatal alloimmune thrombocytopenia, neonatal hemochromatosis, and neonatal Kawasaki. The evidence is limited for other conditions (postnatal management of neonatal alloimmune thrombocytopenia, neonatal thrombocytopenia due to maternal autoimmune disease, neonatal infections, primary immunodeficiency, and others. Because of the unclear risk-benefit ratio of using IVIG to treat infectious and immune-mediated diseases, further studies are needed to evaluate IVIG’s efficacy and safety in fetuses and neonates.

## Figures and Tables

**Table 1:** Suggested Clinical Indications of IVIG Use in Fetuses and Neonates.

Alloimmune hemolytic disease of the newborn
Fetal and Neonatal immune-mediated thrombocytopenia (FNAIT and ITP)
Neonatal infections: Sepsis treatment and prophylaxis Enterovirus infection Parvovirus infection COVID-19 related neonatal disease Congenital CMV
Neonatal hemochromatosis (GALD)
Primary immunodeficiency
Neonatal Kawasaki disease
Neonatal lupus

Note: Suggested uses of IVIG in fetuses and neonates. FNAIT: fetal and neonatal alloimmune thrombocytopenia, ITP: idiopathic thrombocytopenic purpura, COVID-19: coronavirus disease 19, CMV: cytomegalovirus, GALD: gestational autoimmune liver disease.
